# Serum CD26 levels in patients with gastric cancer: a novel potential diagnostic marker

**DOI:** 10.1186/s12885-015-1757-0

**Published:** 2015-10-15

**Authors:** Virginia Boccardi, Luigi Marano, Rosaria Rita Amalia Rossetti, Maria Rosaria Rizzo, Natale di Martino, Giuseppe Paolisso

**Affiliations:** 1 Department of Internal Medicine, Surgical, Neurological Metabolic Disease and Geriatric Medicine, Second University of Naples, Piazza Miraglia 2, 80138 Naples, Italy; 2 General, Minimally Invasive and Robotic Surgery, Department of Surgery, “San Matteo degli Infermi” Hospital, ASL Umbria 2, 06049 Spoleto PG, Italy

**Keywords:** Gastric cancer, Biomarker, sCD26, Dipeptidyl peptidase 4

## Abstract

**Background:**

CD26 is an ectoenzyme with dipeptidyl peptidase 4 (DPP4) activity expressed on a variety of cell types. Considering that serum CD26 levels have been previously associated with different cancers, we examined the potential diagnostic value of serum CD26 levels in gastric cancer.

**Methods:**

Soluble serum CD26 levels were measured in pre and postoperative serum samples of 30 patients with gastric cancer and in 24 healthy donors by a specific ELISA kit.

**Results:**

We found significantly lower serum CD26 levels in patients with gastric cancer (557.7 ± 118.3 pg/mL) compared with healthy donors (703.4 ± 170.3 pg/mL). Moreover patients with HER2 positive tumors had significantly lower CD26 serum levels (511.8 ± 84.8 pg/mL) compared with HER2 negative tumors (619.1 ± 109.9 pg/mL*, p* = 0.006). A binary logistic model having gastric cancer as the dependent variable while age, gender, CEA, CA19.9 and CD26 levels as covariates, showed that CD26 serum levels were independently associated with gastric cancer presence. Indeed after 3 months from surgery serum CD26 levels significantly increased (700.1 ± 119.9 pg/mL vs 557.7 ± 118.3 pg/ml) in all patients (t = −4.454, *p* < 0.0001).

**Conclusions:**

This is a preliminary study showing that the measurement of serum CD26 levels could represent an early detection marker for gastric cancer.

## Background

Gastric cancer, despite its decreasing incidence, represents one of the major health problem worldwide and the fifth most common type of cancer [1]. Gastric cancer is a silent disease frequently diagnosed in advanced stages, which is responsible for its elevated mortality especially among the elderly population where the incidence is significantly higher [2, 3]. Advances in technology have allowed the development of several methods to understand the mechanisms underlying gastric carcinogenesis, resulting in the identification of a large number of molecular targets that can be used as biomarkers with diagnostic and prognostic potentials. Recent studies have identified CD26/ dipeptidyl peptidase 4 (DPP4) as a gene that affects the invasiveness of many tumor cells [4–8] and it is consistently associated with cancer. CD26/dipeptidyl peptidase 4 is a widely expressed cell surface peptidase that exhibits a complex biology with three different functions: adenosine deaminase (ADA) binding, serine peptidase activity, and extracellular matrix (ECM) binding. CD26 may be cell membrane-anchored or in a soluble form, occurring in the serum (sCD26). Cell-associated CD26 is widely expressed on T cells, B cells, natural killer cells, endothelial cells and epithelial cells. The different biological activities of CD26 and its ubiquitous expression may reflect its diverse, sometimes opposing functions in physiological and pathological settings [9].

A deficiency in solubilized CD26 was reported in total homogenates of tumors of colon, kidney, lung and liver. On the contrary, cell-surface CD26 expression has been correlated with disease aggressiveness of T and B cell lymphomas and leukaemias, follicular cell-derived thyroid carcinomas and basal cell carcinomas [10]. In addition, serum DPP4 levels is increased in patients with hepatic cancer and decreased in patients with blood, solid and oral cancer [10, 11]. Recently, CD26 has been identified as a serum marker for colorectal cancer detection [11–13] as well as a prognostic factor able to promote human colorectal cancer metastasis [14], while to best of our knowledge no study investigated the role of serum CD26 in gastric cancer. Only a previous study suggested that CD26 expression level in surgical samples may be considered a reliable biomarker of malignant GISTs of the stomach characterized by distinct clinical, genetic, and histopathological features [15]. Considering that non-invasive biological serum markers would be of great benefit for screening, we aimed at investigating the potential role of CD26 serum levels as a diagnostic marker for gastric cancer detection.

## Patients and methods

### Patients

Preoperative blood samples were collected between January 2013 and October 2014 from 30 patients with histologically documented gastric adenocarcinoma who were candidates for surgical treatment either with curative or palliative intent. Exclusion criteria were previous abdominal radiotherapy, preoperative chemotherapy, diabetes, history of recent immunosuppressive therapy or immunological and hematologic disorders, ongoing infection.

Tumor location, size, Lauren’s type, degree of differentiation, pTNM as well as stage according to the 7^th^ edition of the American Joint Committee on Cancer (AJCC)/Union for International Cancer Control (UICC) tumor, node, metastasis (TNM) staging system [16], oncological radicality of resection and HER2 has been examined.

The control group consisted of 24 healthy blood donors. To avoid bias individuals with evidence of acute inflammatory or infectious diseases, diabetes, immunologic or hematologic disorders have been ruled out. Clinical information was obtained by routine laboratory analyses, history and physical examination. After a clear explanation of the potential risks of the study, all subjects provided written informed consent to participate in the study, which was approved by the ethical committee of the Second University of Naples.

### Analytical methods

Blood samples were collected in the morning after the participants had been fasting for at least 8 h. The drawn blood was allowed to coagulate at room temperature and centrifuged at 2000 g for 15 min. The sera were stored at −85 °C until used.

### Determination of CD26 serum levels

The concentration of serum CD26 were analyzed using a specific immunoassays (Human CD26/DPP4 ELISA Kit, Boster biological technology, Pleasanton, CA) ELISAs were performed according to the manufacturer’s instructions: mean values of duplicated measurements were calculated and a sigmoid-shaped standard curve was determined by simultaneously analyzing a dilution series of standard samples.

### Determination of CEA and CA19.9 serum levels

CEA and CA19.9 levels were analyzed in serum by specific ELISA kits according to manufacturer’s instructions. The cut-off were < 5 ng/ml for CEA and <37 U/ml for CA19.9.

### Determination of HER2 expression in cancer tissues

HER-2 status was tested by immunohistochemistry (IHC) on all tumor samples of evaluated patients while in equivocal (2+) samples fluorescence in situ hybridization (FISH) was performed. Tumor samples immune-stained on 3‑μm thick sections that were mounted on silane‑coated slides using HercepTest™ (Dako, Glostrup, Denmark). Each immune-stained section was evaluated according to ToGA trial criteria: no reactivity or membranous reactivity < 10 % of tumor cells (0+, negative); faint incomplete membranous reactivity in ≥ 10 % of tumor cells (1+, negative); weak to moderate complete basolateral or lateral membranous reactivity in ≥ 10 % of tumor cells (2+, equivocal); strong complete basolateral or lateral membranous reactivity in ≥ 10 % of tumor cells (3+, positive). The FISH test (pharmDx; Dako, Glostrup, Denmark) was performed in all cases of equivocal results (IHC 2+). Gene amplification was recorded when the HER2 and centromere probe 17 (CEP17) signal ratio was >2.0.The HAC samples were tested using polyclonal rabbit anti-human α-1-fetoprotein (clone A000802-29) and monoclonal mouse anti-human hepatocyte (Clone OCH1E5) (Dako, Glostrup, Denmark). Tumors were classified as negative when the HER2:CEP17 ratio was < 1.8 and positive when the HER2:CEP17 ratio was ≥ 2.2. If the HER2:CEP17 ratio was ≥ 1.8 but < 2.2 the specimen was considered equivocal.

### Calculations and statistical analyses

The observed data are normally distributed (Shapiro-Wilk W-Test) and presented as means ± Standard Deviation (SD). The analyses were performed with Chi-square, paired t test or unpaired t test where appropriated. A cut off value for CD26 was determined using receiver operating characteristics (ROC). Sample size calculation was estimated on an IBM PC computer by GPOWER software. The resulting total sample size, estimated according to a global effect size of 25 % with type I error of 0.05 and a power of 80 % was 57 cases. All *p* values presented are 2-tailed and a *p* ≤ 0.05 was chosen for levels of significance. Statistical analyses were performed using SPSS 17 software package (SPSS, Inc., Chicago, IL).

## Results

### CD26 levels in serum of patients with gastric cancer and healthy donors

**Fig. 1:**
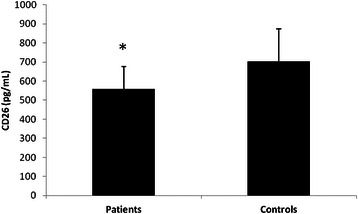
CD26 serum levels in patients with gastric cancer (*n* = 30) and in control healthy individuals (*n* = 24). Patients serum CD26 levels: 557.7 ± 118.3 pg/mL; control serum CD26 levels: 703.4 ± 170.3 pg/mL. *p = 0.001 by unpaired t test

The serum CD26 levels were measured in 24 healthy subjects (14 men and 10 women) with a median age of 63.5 ± 11.1 years. The study group included 30 patients with gastric cancer (16 men and 14 women) with a median age of 66.9 ± 10.2 years. The two groups did not differ in age (*p* = 0.262) and gender (*χ*^2^ = 0.053, *p* = 0.519). The mean serum level of CD26 in patients with gastric cancer was 557.7 ± 118.3 pg/mL, significantly lower than that in healthy individuals (703.4 ± 170.3 pg/mL, *P* = 0.001) (Fig. 1). No difference in CD26 levels were found among gender (*p* = 0.392) in all population study. No correlation was found between CD26 serum levels and age (*r* = −0.178, *p* = 0.232).

### Serum CD26 levels and clinicomorphologic tumors characteristics

Table 1 provides clinicopathologic data for all patients with gastric cancer (n = 30). The relationships between serum CD26 levels and tumor location, size, Lauren’s type, degree of differentiation, pTNM, stage, oncological radicality of resection and HER2 expression were examined (Table 1). No differences in serum CD26 levels were found among all variable described except for HER2 expression: patients with HER2 positive tumors had significantly lower CD26 serum levels (511.8 ± 84.8 pg/mL) compared HER2 negative tumors (619.1 ± 109.9 pg/mL*, p* = 0.006). Patients with positive lymph node metastasis had lower CD26 levels, however such a difference did not reach the statistical significance. Regarding other preoperative markers, CEA and CA19.9 were determined in all patients. A positive correlation between CEA and CA19.9 levels were found (*r* = 0.515, *p* = 0.004). The cut off were determined for both markers according to previous literature: 7 (23.3 %) (*χ*^2^ = 0.900, *p* = 0.279) patients resulted over the cut-off for CA19.9 and 4 (12 %) (*χ*^2^ = 0.279, *p* = 0.471) patients resulted over the cut-off for CEA. The clinical and morphologic tumors characteristics were also studied according to the positive for each of these clinical markers (data not shown) and only HER2 positive tumors were associated with a positive value for CEA (*χ*^2^ = 4.971, *p* = 0.042). No significant correlation between CD26 and other studied markers were found.

**Table 1:** Clinicopathologic data for all patients with gastric cancer (*n* = 30)

	Patients	sCD26 levels (pg/mL)	*p* value
*Tumor location n (%)*			
Upper	6 (20 %)	556.1 ± 77.2	0.986
Middle	11 (36.6 %)	558.0 ± 133.8	
Lower	13 (43.4 %)	564.2 ± 108.5	
*Tumor size n (%)*			
<3 cm	15 (50 %)	569.1 ± 101.2	0.800
>3 cm	15 (50 %)	558.5 ± 121.6	
*Lauren’s Type n (%)*			
Intestinal	20 (60 %)	564.8 ± 94.8	0.934
Diffuse	10 (40 %)	651.0 ± 145.9	
*Differentation n (%)*			
well	4 (13.3 %)	556.3 ± 95.4	0.681
moderately	20 (66.7 %)	553.9 ± 120.0	
poorly	6 (20 %)	559.8 ± 107.2	
*Tumor status n (%)*			
T1a	0	-	0.781
T1b	2 (6.6 %)	556.3 ± 0	
T2	6 (20 %)	563.4 ± 88.1	
T3	12 (40 %)	543.5 ± 146.9	
T4a	7 (23.3 %)	636.7 ± 0	
T4b	3 (10 %)	579.6 ± 88.9	
*Lymph node metastasis n (%)*			
N0	12 (40 %)	602.8 ± 120.1	0.113
N1	8 (40 %)	511.3 ± 50.6	
N2	4(6.6 %)	447.7 ± 109.2	
N3a	2 (10 %)	660.2 ± 0	
N3b	4 (3.4 %)	636.7 ± 0	
*Metastatic disease n (%)*			
M0	26 (86.7 %)	562.0 ± 114.7	0.660
M1	4 (13.3 %)	586.2 ± 83.5	
*Stage n (%)*			
Ia	1 (3.4 %)	546.1 ± 26.5	0.513
Ib	4 (13.3 %)	587.2 ± 81.3	
IIa	2 (6.6 %)	603.3 ± 144.3	
IIb	1 (3.4 %)	626.8 ± 101.8	
IIIa	8 (26.6 %)	587.1 ± 59.7	
IIIb	5 (16.7 %)	613.8 ± 150.4	
IIIc	5 (16.7 %)	564.8 ± 119.7	
IV	4 (13.3 %)	518.9 ± 70.4	
*Resection type n (%)*			
R0	25 (83.3 %)	562.0 ± 114.7	0.660
R1-2	5 (16.7 %)	586.2 ± 83.5	
*HER2 n (%)*			
positive	15 (50 %)	511.8 ± 84.8	0.006
negative	15 (50 %)	619.1 ± 109.9	

### Diagnostic efficiency of CD26 preoperative serum levels

**Fig. 2:**
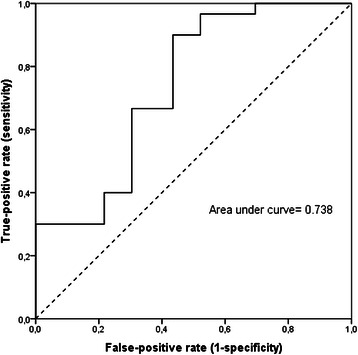
ROC curve for sCD26. ROC curve for sCD26 showed an area under the curve of *C* = 0.738 with SE = 0.071 and 95 % CI from 0.598–0.877. The best cut-off that maximizes (sensitivity + specificity) was 465.8 pg/mL

A binary logistic model having gastric cancer as the dependent variable while age, gender, CEA, CA19.9 and CD26 levels as independent variables, showed that CD26 serum levels were independently associated with gastric cancer presence (Table 2). ROC curve for CD26 showed an area under the curve of *C* = 0.738 with SE = 0.071 and 95 % CI from 0.598–0.877 (Fig. 2). The best cut-off that maximizes (sensitivity + specificity) was 465.8 pg/mL. At this level, the sensitivity was 0.90 and specificity was 0.43. At this established cut off level 8 (26.6 %) patients resulted behind the cut-off (*χ*^2^ = 7.224, *p* = 0.007) showing higher diagnostic efficiency compared CEA and CA19.9. After 3 months from surgery CD26 levels significantly increased (700.1 ± 119.9 pg/mL vs 557.7 ± 118.3 pg/mL) in all patients (t = −4.454, *p* < 0.0001).

**Table 2:** Binary logistic regression analysis with gastric cancer as the dependent variables in all population study (*n* = 54)

	B	Odds Ratio	95 % CI	*p*
Gender	−0.188	0.828	0.220–3.123	.828
Age	0.019	1.019	0.957–1.086	.551
CD 26	−0.007	0.993	0.988–0.998	.003
CEA	0.837	2.308	0.255–20.928	.457
CA19.9	0.507	1.660	0.277–9.963	.579

Gender expressed as F = 0 an M = 1

## Discussion

The major findings of our investigation are: i) patients affected by gastric cancer have significantly lower serum CD26 levels compared with healthy subjects ii) CD26 serum levels are associated with gastric cancer presence iii) CD26 measurement shows higher diagnostic efficiency compared CEA and CA19.9 in gastric cancer iiii) patients with HER2 positive tumors have significantly lower serum CD26 levels compared with the HER2 negative counterpart.

Gastric cancer represents the third most common cause of cancer-related death in the world and most patients present advanced disease at diagnosis making its treatment very intricate [1–3]. It is widely accepted that early diagnosis and treatment are keys for better clinical outcome in patients with gastric cancer [2, 3].

CD26 is a multifunctional cell surface glycoprotein with intrinsic dipeptidyl peptidase 4 activity particularly expressed on epithelial cells and lymphocytes [9, 17, 18]. However, CD26 also exists as a soluble circulating form in plasma and its role it is still unknown. Cell surface proteases participate in malignant transformation and cancer progression by promoting invasion and metastasis. Sometimes they can also gave an opposite effect, this is the case of CD26 [11, 17]. In fact, surface CD26 expression is lost or altered in melanoma, hepatocellular carcinoma and colon cancer cells [10]. It has been suggested that CD26 with its enzymatic activity is able to degrade growth factors required for survival and invasiveness of tumor cells [12]. Also serum CD26 is substantially dysregulated in various cancers: serum CD26 levels are increased in patients with hepatic cancer and decreased in patients with blood, thyroid and oral cancers [10, 11]*.* Recently, CD26 has been identified as a serum marker for colorectal cancer detection and prognostic factor [12–14] while to best of our knowledge no study investigated the role of serum CD26 in presence of gastric cancer.

With this preliminary study we found that patients affected by gastric cancer have lower levels of circulating serum CD26 compared with healthy controls, thus representing a powerful biomarkers of gastric cancer. We found a lack of any association of sCD26 levels with tumor localization, size, type, differentiation, TNM, stage or lymph node metastases. Accordingly Cordero and colleagues [12, 13] did not found any relationship between the levels of sCD26 and the Dukes’ stage classification in patients affected by colorectal cancer. These data collectively suggest the potential usefulness of this molecule for early diagnosis of gastric cancer. The regression analysis showed that lower sCD26 levels were associated to gastric cancer presence independent of age, gender and others tumors biomarkers (CA19.9 and CEA). This findings further support the relevance of CD26 as a new diagnostic marker for gastric cancer with higher efficiency compared with other available biomarkers as shown by the ROC curve. Whether lower CD26 serum levels are associated with lower or higher tumor surface expression is unknown and may represent a limitation of this study. However, previous evidence are showing that impairment in sCD26 in colorectal cancer does not seem to be originated by alteration of CD26 on tumour cells [11–13]. Thus, we can speculate that the drop in serum CD26 levels may be related to a dysfunction in the immune system status in patients with gastric cancer. In fact, a cross-talk between the lymphoid lineage and malignant tumors in vivo have been long discussed and some data about the immune defective antitumor response in many cancers, such as colonic, have been described before, including a defect in IL-12 production [19], which is a well-known CD26 up-regulator on T cells [20]. Again in oral cancer patients, in which around a 50 % decrease in serum CD26 activity has been reported, a correlation between sCD26 and CD26+ T was found, and the mount of CD26 in T lymphocyte plasma membranes were significantly lower than in healthy subjects [21]. Thus, even in the gastric cancer, the hypothetic role of sCD26 in crosstalk between the immune system and carcinogenesis, cannot be ruled out. Further studied are needed to test such an hypothesis and to collect the lymphocyte count, subset distribution and other immune parameters in patients with gastric cancer. Interestingly recent studies are showing that sCD26 therapy enhances the immune function in some pathological conditions such as AIDS [22] and it might be interesting to analyze if gastric cancer patients may well benefit from exogenous sCD26 treatment.

Our most important finding is that lower serum CD26 levels were found particularly in sera of patients with HER2 positive tumors. Upon the increased knowledge of breast cancer cells molecular pathways, the biological feature of gastric cancer is becoming more clear and particular attention should be paid to the identification of the human epidermal growth factor receptor-2 (HER2) amplified gastric cancer subtype. This latter accounts for 10–38 % of all gastric cancers, with an higher prevalence in tumors from the upper third of the stomach than in those located in more distal areas, as well as in Lauren’s intestinal type than in diffuse-type gastric cancer [23]. HER2 protein overexpression on the gastric cancer cells’ surface with its enhanced and prolonged signals influence particularly the carcinogenesis processes determining distinctive clinic-pathological phenotype characterized by acquisition of advantageous properties for excessive and uncontrolled cell growth, identifying a distinctive gastric cancer entity [24]. From a prognostic perspective, even though the negative prognostic effect of HER2 positivity in breast cancer is well assessed, the relationship between HER2 status and prognosis of gastric cancer patients remains controversial [25]. Nevertheless the administration of standard chemotherapy with Trastuzumab, a monoclonal antibody that binds to the extracellular domain of the HER2 receptor blocking its downstream signaling, shows a clinically meaningful improvement in overall survival for patients with HER2-positive advanced gastric cancer [26]. The lower serum CD26 levels in patients with HER2 positive tumors suggests an attractive linking mechanism between innate effector cells/T-cells immunity and the anti-tumoral effects. Supporting this, it has been demonstrated that CD26 is a potential target for amino boronic dipeptide PT-100, a dipeptidyl peptidase (DPP) inhibitor, which is able to augment the effect of trastuzumab on the growth inhibition of HER2 positive carcinoma cell lines in animal models [27].

Indeed, establishing the diagnosis at an early stage in gastric cancer, with a simple biochemical index, is a current subject of research in clinical oncology. The CEA levels are the marker of reference in this neoplasia, although not recommended as diagnostic test [28]. Our results revealed that sensitivities of sCD26 were higher at different specificity levels than those of CEA or CA 19.9 as well as efficiency. Indeed, the fact the CD26 levels significantly increase after surgery may indicate that the sCD26 serum level may be used also as prognostic marker. However further studies are needed to validate such an hypothesis.

## Conclusions

Using novel biomarkers to early gastric cancer detection may potentially decrease mortality and medical costs. Preoperative sCD26 level may represent an useful and easy biomarker for early detection of gastric cancer. Indeed the finding that patients with HER2 positive tumors have lower sCD26 levels may have clinical potential in gastric cancer management improving the effect of drugs on the growth inhibition of HER2 positive cancers.

## Abbreviations

DPP4: Dipeptidyl peptidase 4
IHC: Immunohistochemistry
FISH: Fluorescence in situ hybridization
ROC: Receiver operating characteristics
